# Polyphenols Coordinated with Cu (II) in an Aqueous System Build Ion-Channel Coatings on Hair Surfaces

**DOI:** 10.3390/ma16041333

**Published:** 2023-02-04

**Authors:** Lei Jin, Daemyoung Yun, Wei Zhang, Jinsung Lee, Hongchul Shin, Donghyuk Kim, Tae-Bong Kang, Hyung-Sik Won, Hohyoun Jang, Whangi Kim

**Affiliations:** 1 Department of Applied Chemistry, Konkuk University, Chungju 27478, Republic of Korea; 2 Suan Hyangjang Co., Ltd., Suan bd 204, Jungwon-gu, Seongnam 13204, Republic of Korea; 3 Department of Applied Life Sciences, Graduate School, BK21 Program, Konkuk University, Chungju 27478, Republic of Korea; 4 Department of Biotechnology, College of Biomedical & Health Science, Konkuk University, Chungju 27487, Republic of Korea

**Keywords:** hair dye, pyrogallol, gallic acid, ion channel, coating, aqueous system

## Abstract

Recently, developments in the field of cosmetics have led to a renewed interest in hair dyeing. However, damage to the hair during the dyeing process has increased hesitation in attempting hair dyeing. As a result, hair dyes with minimal side effects have been in constant demand, and are being developed. In this study, natural-extract polyphenols, pyrogallol, and gallic acid are coordinated by CuCl_2_ in a NaCl aqueous solution to form an oligomer, which creates an ion-channel coating on the hair surface to protect it. This work attempts to develop fast, simple, and damage-free hair-dye ingredients based on pyrogallol and gallic acid. The morphology and elements of polyphenols coated on hair are characterized. The results reveal that the hair is dyed with the polyphenol-based dye reagent successfully. Moreover, the thickness of the dyed hair continuously rises ten times after dyeing. The tensile strength of the dyed hair is also measured, showing an upward and downward trend. These results reflect the fact that pyrogallol and gallic acid are considered to be the essential and functional polyphenols, and can build ion blocks on hair, which can create new multifunctional coating materials.

## 1. Introduction

With the advancement of globalization, hair dye has become more and more popular among all cultures and ethnicities, desirable not only in natural colors but also in colors beyond individuals’ genetic predispositions [1,2,3]. As a result, hair dyeing has become one of the most prosperous industries in cosmetics to date [4,5,6]. Human hair has a simple structure that is mainly composed of protein. In the hair shaft, keratin cells are the most abundant element, consisting of the pigment melanin [7,8]. Melanin determines the color of human skin, hair, and eyes [9,10,11,12,13]. As time goes on and the amount of melanin decreases, hair color also changes to gray or even white. Generally, the average age for white hair onset is the mid-30s, increasing rapidly from 50 years old onwards [14]. Whether for maintaining natural hair color or changing the hair color from its natural color, hair dyeing is undoubtedly the most intuitive and convenient method.

Most commercial chemical dyes contain bleaching agents such as ammonia and peroxide [15,16,17]. During the hair-dyeing process, bleaching agents first damage the outermost cuticle layer of the hair and push chemical colors such as p-Phenylenediamin (PPD), o-Phenylenediamine (OPD), and m-Phenylenediamine (MPD) to enter the cortex layer [18,19,20,21,22,23,24]. The bleaching agents fade the pigment melanin and further react with chemical colors, creating a new color and stocking inside the hair. This process can usually bring about a permanent hair color but can result in hair damage, unpleasant odor, and even allergic reactions [25,26]. By contrast, natural dyes color hair by coating the hair shaft and presenting a substitute, placing more emphasis on hair-dye safety. They maintain the thickness of the hair and stick as a semi-permanent color. Polyphenols, pyrogallol, and gallic acid can be extracted from tea leaves, gallnuts, and other plants [27,28,29]. Modern hair dyes use pyrogallol and gallic acid, revolutionary coating methods with different chemical structure mechanisms that are safe compared with the existing standard dyes [30,31]. Mohamed El-Wekil et al. [32] reported that the hydroxyl groups in pyrogallol complexed with Cu(II)Cl_2_ and formed a coordination complex between Cu and a catechol moiety. The formation showed a yellow color at a 345 nm absorbance colorimetric determination.

In this work, we prepare a polyphenols-based hair dye polymerized by CuCl_2_ in a NaCl aqueous solution. Cu(II) ions can complex with hydroxyl groups to create a Cu–O coupling and form an ion channel on the hair surface, thus protecting the cuticle layer and further decreasing the hair damage during the dyeing process [32,33,34]. Coating deposition on the hair continues layer-by-layer with Cu(II) ions leading to the controllable thickness in the growth of the hair. We characterize the morphology and element contribution via field emission scanning electron microscopy (FE-SEM) and energy-dispersive X-ray spectroscopy (EDS). The measurement results suggest that the Cu, Cl, and Na elements distributed on the hair surface and the amount of elements increases as the dyeing time increases. Furthermore, the cross-sectional EDS results show that only a few Cu and Cl elements were detected, indicating that the hair dye did not break the hair surface. The thickness and tensile strength of the hair dyed with polyphenols and amino-based commercial products are also compared. However, we find that the tensile strength of the polyphenols-based dyed hair is a little lower than that of the commercial-product dyed hair after dyeing ten times; this is because when the thickness of the hair surface is increased, the tensile strength of the hair may decrease.

## 2. Experiment

### 2.1. Materials

Pyrogallol (ACS reagent), gallic acid (ACS reagent, ≥98.0%), cupric (II) chloride (CuCl_2,_ powder, 99%), L-arginine (reagent grade, ≥98%), and sodium chloride (NaCl, ACS reagent, ≥99.0%) were purchased from Sigma-Aldrich (Seoul, Republic of Korea). All reagents were used as received, without further purification. Human natural (noncommercial) white-hair samples were supplied by Suan Hyangjang Co., Ltd. (Seongnam, Republic of Korea). Dulbecco’s Modified Eagle Medium (DMEM), fetal bovine serum, and streptomycin were purchased from Gibco of Thermo Fisher Scientific (Seoul, Republic of Korea). The 3-(4,5-dimethylthiazol-2-yl)-2,5-diphenyl-2H-tetrazolium bromide was purchased from Sigma-Aldrich (Seoul, Republic of Korea).

### 2.2. Synthesis of Gallic Acid and Pyrogallol Oligomer

To prepare the gallic-acid-based hair dye, gallic acid (17.6 mmol) was first dissolved in H_2_O (100 mL) at room temperature (RT), and was then mixed with CuCl_2_ (23.5 mmol) and NaCl (29.9 mmol) (in 100 mL H_2_O) solution for 5 min to obtain the gallic-acid-based hair dye. The pH of the dye reagent was adjusted to 5, using L-arginine (1.14 mmol). The pyrogallol-based oligomer was prepared via the same method but with a slightly different weight ratio, which is presented in Supplementary Figure S1. 

### 2.3. Hair-Dyeing Process

To carry out hair dyeing, the white-hair samples were soaked in a mixture solution of gallic acid and CuCl_2_ agents. The hair samples were rubbed until foam generation and were then kept in this state for 2 min. Finally, the dyed hair was washed with shampoo twice and with running water several times until the water was neutral. The hair samples were obtained after 30 s of hair drying. The hair samples were prepared with different dyeing times and named as S-n, where ‘n’ represented the hair-dyeing time. The hair samples dyed using commercial products were dyed in the same conditions, while following the product instructions.

### 2.4. Characteristics of Dyed Hair

The chemical structure was monitored by Fourier-transform infrared spectroscopy (FT-IR) using a Nicolet iS5 (Waltham, US) from 4000 to 500 cm^–^1 with a spectral resolution of 4 cm^−1^. FT-IR studies analyzed solid states of Gallic acid and dyed regent. The FE-SEM and energy-dispersive X-ray-spectroscopy (EDS) analyses were performed on a JSM-6700F (JEOL, Japan) with an accelerating voltage of 15.0 kV to investigate the surface morphology and elemental composition of materials. The uniaxial tensile tests were conducted on an LFV-250HH (Walter + Bai AG, Switzerland) testing machine with a crosshead speed of 5 mm min^−1^. At least three specimens were tested for each composition. All the dyed-hair samples were cut with liquid nitrogen to ensure cross-sectional integrity.

HaCaT keratinocytes were cultured in Dulbecco’s Modified Eagle Medium (DMEM) complemented with 10% fetal bovine serum, 50 U/mL penicillin, and 50 µg/mL streptomycin under a humidified atmosphere of 5% CO_2_ at 37 °C [35]. HaCaT cells were seeded in 24-well plates at 7 × 104 cells/mL density. The next day, the dyed and non-dyed hairs were loaded onto the cells and cultured for 24 h. The microscopic observation was performed using an OLYMPUS CKX53, and 3-(4,5-dimethylthiazol-2-yl)-2,5-diphenyl-2H-tetrazolium bromide solution was added at a 500 ng/mL final concentration. The plate was then further incubated for 4 h. The medium was removed, and the insoluble formazan was dissolved in 400 μL DMSO. Dissolved formazan was transferred to a 96-well plate (100 μL each well) to measure the absorbance. The absorbance was determined at 540 nm with a reference wavelength of 630 nm in a microplate reader (Thermo scientific, MULTISKAN GO). The cell viability was calculated as follows: % Viability = A 450 − A 630 of test cells/A 450 − A 630 of non-treated control cells × 100.

## 3. Results and Discussion

**Figure 1:**
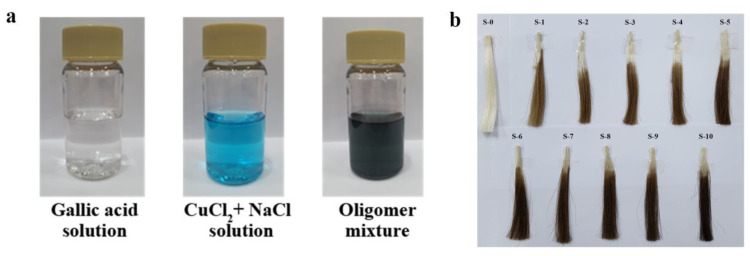
Photographs of (**a**) gallic acid, CuCl_2_ + NaCl, oligomer-mixture solution mixture, and (**b**) the hair samples.

Gallic acid, a natural compound, is classified as a phenolic acid distributed in tea or plant leaves. It can be dissolved in organic solvents such as alcohol, ether, acetone, and water. Thus, most hair dyes consist of gallic acid and use organic compounds as a solvent. This study dissolved gallic acid in water, and the hair-dye reagent was prepared in an aqueous system. The prepared solutions in Figure 1 show that (a) the transparent gallic-acid aqueous solution mixed with blue-green CuCl_2_ and NaCl solution produced a dark-green oligomer. The prepared oligomer mixture was used to carry out hair dyeing. As displayed in Figure 1b, the uncoated hair S-0 was white, and turned dark brown after dyeing. The color characteristics of dyed hairs were changed in the order of white, earthy yellow, light brown, grayish brown, brown, and dark brown as the amount of dyeing time increased.

**Figure 2:**
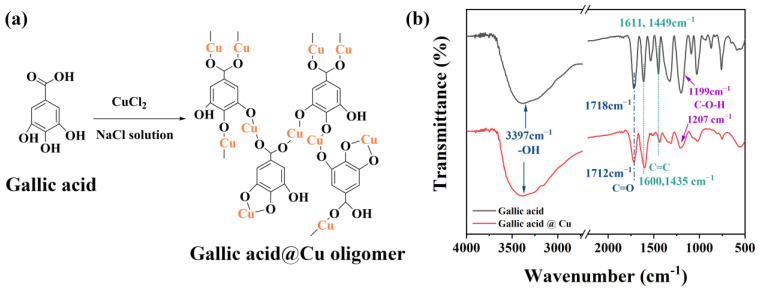
(**a**) Illustration of chemical structure, (**b**) FT-IR spectrum of gallic acid and after complex with Cu(II).

Moreover, the chemical structure of the oligomer was confirmed by FT-IR at RT. Figure 2a presents the chemical structure after gallic acid coordinated with Cu(II), and the FT-IR result is shown in Figure 2b. The characteristic peak -OH of gallic acid shows at 3397 cm^−1^; C=O double-bond stretching can be found at 1718 cm^−1^, and the aromatic C=C stretching is present at 1600 and 1435 cm^−1^ as a double peak. C-O-H bending absorption is detected at 1199 cm^−1^ for gallic acid. In the case of the gallic-acid oligomer, the main changes in the spectrum were that: (1) the C=O double-bond stretching moved to a lower wavenumber (1718 to 1712 cm^−1^); (2) the C=C double bond shifted to the right, from 1661 and 1449 to 1600 and 1435 cm^−1^, respectively; and (3) the peak of C-O-H bending decreased dramatically, due to the conjugation between the hydroxide group and the metal of Cu causing small frequency shifts [36,37,38,39].

**Figure 3:**
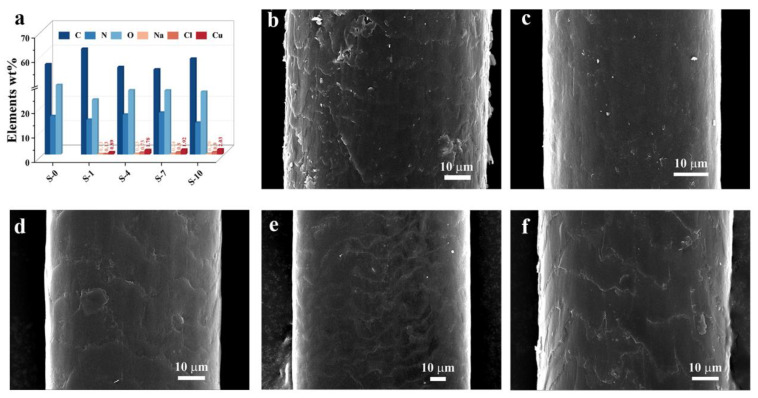
(**a**) Elements distributed on the hair surface; (**b**–**f**) FE-SEM images of the hair samples for S-0, S-1, S-4, S-7, and S-10.

EDX and FE-SEM were used to further investigate the distribution of the elements on the hair and confirm the hair-sample morphology. The resulting EDX values investigating the elements distributed on the hair surface are summarized in Figure 3a, and the original data can be found in Supplementary Figure S2. Figure 3a shows that the blank hair sample (S-0) consisted of C, N, and O, due to the hair mainly consisting of protein. Moreover, Na, Cl, and Cu elements appeared on the hair surface after dyeing once, and the content increase trend was in direct proportion to the number of times dyed. Furthermore, there were no considerable changes to the amount of C, N, or O, revealing that the elements coated the hair layer by layer. The FE-SEM images of hair samples in Figure 3b–f show that the surfaces of the hair became smoother as the number of dyeing times increased, indicating that the hair was coated with the dye reagents.

**Figure 4:**
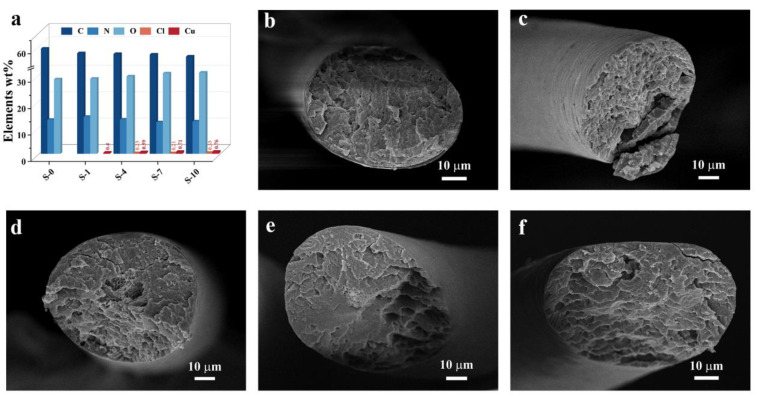
(**a**) Elements distributed on the hair cross-section; (**b**–**f**) cross-sectional SEM images of the hair samples for S-0, S-1, S-4, S-7, and S-10.

Furthermore, the cross-section element distribution and morphology were identified using the same method, and the results are presented in Figure 3. Figure 4a shows that all the hair samples were composed of C, N, and O. Unlike the surface-element measurement results, Na was not found in the cross-section of the dyed hair, and only extremely low quantities of Cl and Cu were detected. Additionally, the cross-sectional SEM images demonstrated in Figure 4b–f revealed no damage on the hair surface, and the hair was thoroughly coated with hair dye. The row data of the element-distribution proportions are displayed in Figure S3.

**Figure 5:**
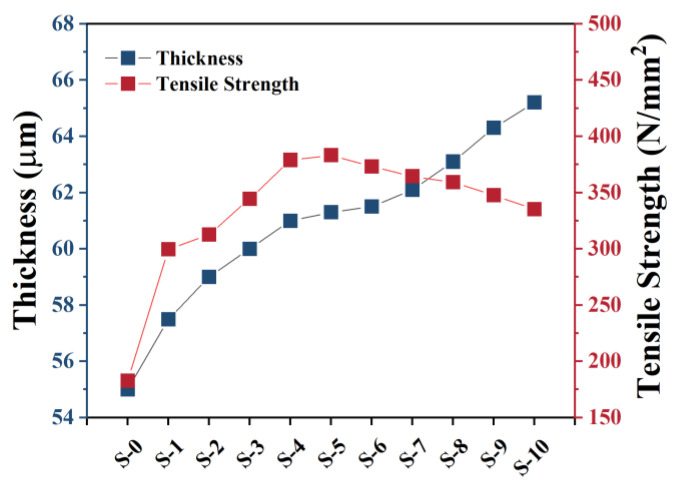
Thickness and tensile strength of hair before and after dyeing process.

To further study the hair dye on the hair, the thicknesses and tensile strengths of the hair samples were also examined. The deep-blue line in Figure 5 represents the thickness of the hair before and after hair dyeing. The thickness of the hair clearly crept up slowly, suggesting that every single dyeing process may have formed a coating layer on the hair surface, increasing the hair thickness. Similarly, the hair’s tensile strength (red line) also increased with the number of times it was dyed. However, the hair’s tensile strength climbed to 375 N/mm^2^ and then changed to a dropping tendency. This result may be attributed to the hair dye increasing the hair thickness to a certain degree, with a too-high thickness decreasing the hair toughness and tensile strength of the hair.

**Figure 6:**
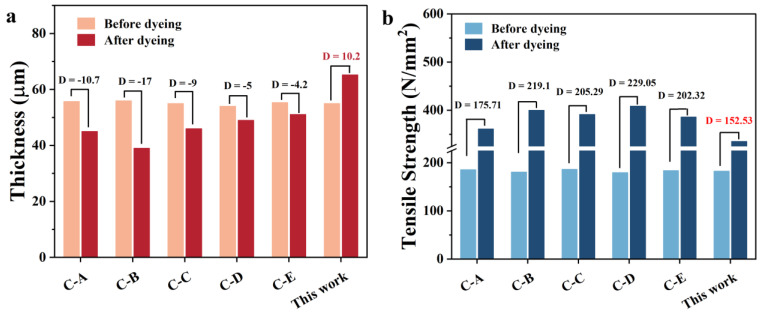
Comparing (**a**) thicknesses and (**b**) tensile strengths of dyed hair using commercial products and gallic-acid-based dye reagents.

In addition, to better study the effect of aqueous and amine-based hair dye on the dyed hair, the thicknesses and tensile strengths of hair dyed using commercial amine-based products and polyphenols-based reagents were measured. Figure 6a shows the compared thicknesses of the hair samples. All the commercial products decreased the hair thickness to a certain degree, while the thickness value increased when the aqueous gallic-acid hair dye was used. However, the hair samples dyed using commercial amine-based products reflected superior tensile strength, compared with that of the prepared gallic-acid-based dyed hair, indicating that the thickness of hair can affect hair strength directly. Figure 6 indicates that all the hair samples were dyed ten times, and the values are displayed in Table S2.

The hair-dye reagent prepared with pyrogallol was also characterized, and the results are presented in the Supplementary Materials. The pyrogallol-based hair dye demonstrated a similar result to that of the gallic-acid-based hair dye, which was able to coat the hair surface and increase the hair’s thickness.

**Figure 7:**
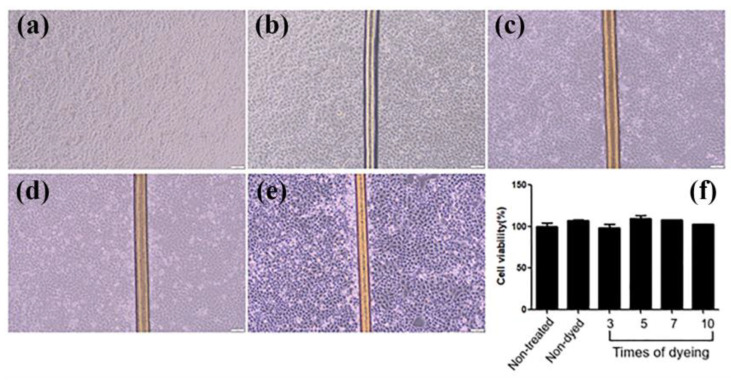
(**a**–**e**) The effects of dyed hair on human-keratinocyte-growth microscopic analysis, and (**f**) MTT assay of human keratinocytes co-cultured with hair for 24 h (magnification 20×).

Moreover, to examine whether dyed hairs affected cell viability, keratinocytes were cultured with hairs dyed at the indicated times, for 24 h. Their microscopic morphology and cell viability were compared to those of cells treated with non-dyed hair. We found that the application of hair barely affected cell growth, including morphological change (Figure 7a–e) and cell viability (Figure 7f). Furthermore, cells could be attached to the dyed hairs and grown on them, indicating that the dye is a non-cytotoxic material for keratinocytes.

## 4. Conclusions

In this study, polyphenols, including pyrogallol and gallic-acid-based hair dyes, were prepared with CuCl_2_ in an aqueous NaCl solution. Cu, Cl, and Na compounds were detected on the dyed-hair surface, and the amount of element also increased as the dyeing times increased. With respect to the cross-sectional element-analysis results, only a small quantity of Cl and Cu was found, indicating that the hair dye coated the hair surface layer by layer, and did not destroy the hair structure. The coating on the hair surface enhanced the thickness of the hair and presented a tensile strength which was little lower than that of commercial products. The ion channel built by Cu (II) in a NaCl aqueous solution with hydroxyl groups on the hair surface further protected the hair from damage during the dyeing processes. A biosafety study further confirmed that the prepared hair dye was safe for hair cells. Based on these results, plant-extracted polyphenols are promising materials for green, safe, and environmentally friendly hair dye.

## Data Availability

Not applicable.

## Supplementary Materials

The following supporting information can be downloaded at: https://www.mdpi.com/article/10.3390/ma16041333/s1, Figure S1: Photographs of pyrogallol, CuCl_2_ + NaCl, and oligomer solution mixture. Figure S2: Elements distributed on the hair surface. Figure S3: Elements distributed on the hair cross-section. Figure S4: Photographs of the hair samples. Figure S5: Thickness and tensile strength of hair before and after dyeing with pyrogallol. Table S1: Comparing thicknesses and tensile strengths of hair dyed with commercial products and with gallic-acid-based dye.

## References

[1] Rust R.C., Schlatter H. (2022). Hair Dyes. Cosmetic Dermatology.

[2] Ali A., Moinuddin A.A., Allarakha S., Fatima S., Ali S.A., Habib S. (2022). Risk of Carcinogenicity Associated with Synthetic Hair Dyeing Formulations: A Biochemical View on Action Mechanisms, Genetic Variation and Prevention. Indian J. Clin. Biochem..

[3] Da França S.A., Dario M.F., Esteves V.B., Baby A.R., Velasco M.V.R. (2015). Types of Hair Dye and Their Mechanisms of Action. Cosmetics.

[4] Guerra-Tapia A., Gonzalez-Guerra E. (2014). Hair Cosmetics: Dyes. Actas Dermo-Sifiliográficas (Engl. Ed.).

[5] Coimbra S.C., Sousa-Oliveira I., Ferreira-Faria I., Peixoto D., Pereira-Silva M., Mathur A., Pawar K.D., Raza F., Mazzola P.G., Mascarenhas-Melo F. (2022). Safety Assessment of Nanomaterials in Cosmetics: Focus on Dermal and Hair Dyes Products. Cosmetics.

[6] De Souza J.C., da Silva B.F., Morales D.A., Umbuzeiro G.D.A., Zanoni M.V.B. (2021). Assessment of the Compounds Formed by Oxidative Reaction between P-Toluenediamine and p-Aminophenol in Hair Dyeing Processes: Detection, Mutagenic and Toxicological Properties. Sci. Total Environ..

[7] Williams T.N., Szymczyk M., Freeman H.S. (2021). In Situ Chelation of Monoazo Dyes in Human Hair Keratin Fibers Using Environmentally Benign Metal Ions. ACS Appl. Bio. Mater..

[8] He X., Wang J.F., Wang Y. (2021). Influence of Cosmetic Hair Treatments on Hair of Methamphetamine Abuser: Bleaching, Perming and Coloring. Ecotoxicol. Environ. Saf..

[9] Agarwal V., Panicker A.G., Indrakumar S., Chatterjee K. (2019). Comparative Study of Keratin Extraction from Human Hair. Int. J. Biol. Macromol..

[10] Cesarini J.P. (1990). Hair Melanin and Hair Color. Hair and Hair Diseases.

[11] Nishikawa N., Tanizawa Y., Tanaka S., Horiguchi Y., Asakura T. (1998). Structural Change of Keratin Protein in Human Hair by Permanent Waving Treatment. Polymer.

[12] Xiao M., Li Y., Allen M.C., Deheyn D.D., Yue X., Zhao J., Gianneschi N.C., Shawkey M.D., Dhinojwala A. (2015). Bio-Inspired Structural Colors Produced via Self-Assembly of Synthetic Melanin Nanoparticles. ACS Nano.

[13] Battistella C., Mccallum N.C., Gnanasekaran K., Zhou X., Caponetti V., Montalti M., Gianneschi N.C. (2020). Mimicking Natural Human Hair Pigmentation with Synthetic Melanin. ACS Cent. Sci..

[14] Sarna T., Burke J.M., Korytowski W., Rózanowska M., Skumatz C.M.B., Zarȩba A., Zarȩba M. (2003). Loss of Melanin from Human RPE with Aging: Possible Role of Melanin Photooxidation. Exp. Eye Res..

[15] Souza J.C., Machini W.B.S., Zanoni M.V.B., Oliveira-Brett A.M. (2020). Human Hair Keratin Direct Electrochemistry and In Situ Interaction with P-Toluenediamine and P-Aminophenol Hair Dye Precursors Using a Keratin Electrochemical Biosensor. ChemElectroChem.

[16] Corrêa G.T., de Souza J.C., Silva J.P., Pividori M.I., Zanoni M.V.B. (2020). Determination of Temporary Dye Basic Red 51 in Commercial Hair Dye, River Water and Wastewater from Hairdressing Salon Using Graphite-Epoxy Composite Electrode Modified with Magnetic Nanoparticles. Microchem. J..

[17] De Souza J.C., Zanoni M.V.B., Oliveira-Brett A.M. (2020). Genotoxic Permanent Hair Dye Precursors P-Aminophenol and p-Toluenediamine Electrochemical Oxidation Mechanisms and Evaluation in Biological Fluids. J. Electroanal. Chem..

[18] Meyer A., Fischer K. (2015). Oxidative Transformation Processes and Products of Para-Phenylenediamine (PPD) and Para-Toluenediamine (PTD)—A Review. Environ. Sci. Eur..

[19] Ko H.-Y., Lin Y.-H., Shih C.-J., Chen Y.-L. (2019). Determination of Phenylenediamines in Hair Colors Derivatizated with 5-(4, 6-Dichlorotriazinyl)Aminofluorescein via Micellar Electrokinetic Chromatography. J. Food Drug Anal..

[20] Hamann D., Yazar K., Hamann C.R., Thyssen J.P., Lidén C. (2014). P-Phenylenediamine and Other Allergens in Hair Dye Products in the United States: A Consumer Exposure Study. Contact Dermat..

[21] Warsi M.S., Habib S., Talha M., Mir A.R., Alam K., Ali A. (2021). Characterization of Human Serum Albumin Modified by Hair Dye Component, 4-Chloro-1, 2-Phenylenediamine: Role in Protein Aggregation, Redox Biology and Cytotoxicity. J. Mol. Liq..

[22] Niu Y., Li Y., Ma F., Zhang M., Chen X., Ye B.-C. (2023). Ratiometric Electrochemical Sensing Platform Based on N-Doped MOF-Derived CoNi/C for the Determination of p-Phenylenediamine in Hair Dyes. Microchim. Acta.

[23] He L., Michailidou F., Gahlon H.L., Zeng W. (2022). Hair Dye Ingredients and Potential Health Risks from Exposure to Hair Dyeing. Chem. Res. Toxicol..

[24] Zhang S., Liu B., Li W., Lin T., Yang H., Pei Y., Gong Z. (2021). Highly Selective and Sensitive Fluorescence Determination of M-Phenylenediamine. Microchem. J..

[25] Venkatesan G., Dancik Y., Sinha A., Kyaw H.M., Srinivas R., Dawson T.L., Bigliardi M., Bigliardi P., Pastorin G. (2021). Development of Novel Alternative Hair Dyes to Hazardous Para-Phenylenediamine. J. Hazard. Mater..

[26] Sampathkumar K., Yesudas S. (2009). Hair Dye Poisoning and the Developing World. J. Emerg.Trauma Shock.

[27] Sileika T.S., Barrett D.G., Zhang R., Lau K.H.A., Messersmith P.B. (2013). Colorless Multifunctional Coatings Inspired by Polyphenols Found in Tea, Chocolate, and Wine. Angew. Chem. Int. Ed. Engl..

[28] Geng H., Dai Q., Sun H., Zhuang L., Song A., Caruso F., Hao J., Cui J. (2020). Injectable and Sprayable Polyphenol-Based Hydrogels for Controlling Hemostasis. ACS Appl. Bio. Mater..

[29] Wang W., Chen Y.-F., Wei Z.-F., Jiang J.-J., Peng J.-Q., He Q.-T., Xu W.-Y., Liu H.-M. (2023). Microemulsion of Cinnamon Essential Oil Formulated with Tea Polyphenols, Gallic Acid, and Tween 80: Antimicrobial Properties, Stability and Mechanism of Action. Microorganisms.

[30] Panwar V., Dey B., Sheikh J.N., Dutta T. (2022). Thermostable Bacterial Laccase for Sustainable Dyeing Using Plant Phenols. RSC Adv..

[31] Jia D., Shen Y., Zhang X., Wang Y., Su R., Qi W. (2022). Colorful Pigments Based on Multicomponent Metal-Phenol Network Nanoparticles for Hair Dyeing. ChemistrySelect.

[32] Ali H.R.H., Hassan A.I., Hassan Y.F., El-Wekil M.M. (2019). Colorimetric and Fluorimetric (Dual-Mode) Nanoprobe for the Determination of Pyrogallol Based on the Complexation with Copper(II)- and Nitrogen-Doped Carbon Dots. Microchim. Acta.

[33] Hung C.H., Chang W.T., Su W.Y., Cheng S.H. (2014). Electrochemical Determination of Pyrogallol at Conducting Poly(3,4-Ethylenedioxythiophene) Film-Modified Screen-Printed Carbon Electrodes. Electroanalysis.

[34] Shin M., Park E., Lee H. (2019). Plant-Inspired Pyrogallol-Containing Functional Materials. Adv. Funct. Mater..

[35] Boukamp P., Petrussevska R.T., Breitkreutz D., Hornung J., Markham A., Fusenig N.E. (1988). Normal Keratinization in a Spontaneously Immortalized Aneuploid Human Keratinocyte Cell Line. J. Cell Biol..

[36] Rocha J.E., Guedes T.T.A.M., Bezerra C.F., Costa M.D.S., Campina F.F., de Freitas T.S., Sousa A.K., Souza C.E.S., Silva M.K.N., Lobo Y.M. (2021). FTIR Analysis of Pyrogallol and Phytotoxicity-Reductive Effect against Mercury Chloride. Environ. Geochem. Health.

[37] Kim B.J., Han S., Lee K.B., Choi I.S. (2017). Biphasic Supramolecular Self-Assembly of Ferric Ions and Tannic Acid across Interfaces for Nanofilm Formation. Adv. Mater..

[38] Devi J., Yadav M., Sharma S., Devi J. (2018). Synthesis and Characterization of Co (Ii), Ni (Ii), Cu (Ii), and Zn (Ii) Complexes of Thiosemicarbazones. Molecules.

[39] Ejima H., Richardson J.J., Liang K., Best J.P., van Koeverden M.P., Such G.K., Cui J., Caruso F. (2013). One-Step Assembly of Coordination Complexes. Science.

