# Supplementary material for: Polyphenols Coordinated with Cu (II) in an Aqueous System Build Ion-Channel Coatings on Hair Surfaces
Source: Materials (Basel). 2023 Feb 4;16(4):1333. doi: 10.3390/ma16041333 (PMC9967149; doi:10.3390/ma16041333)
Supplement: Supplementary file 1 [file materials-16-01333-s001.zip › materials-2162983-supplementary.pdf]

Supplementary Material

Polyphenols Coordinated with Cu (II) in an Aqueous System Build ion Channel Coatings on Hair Surfaces

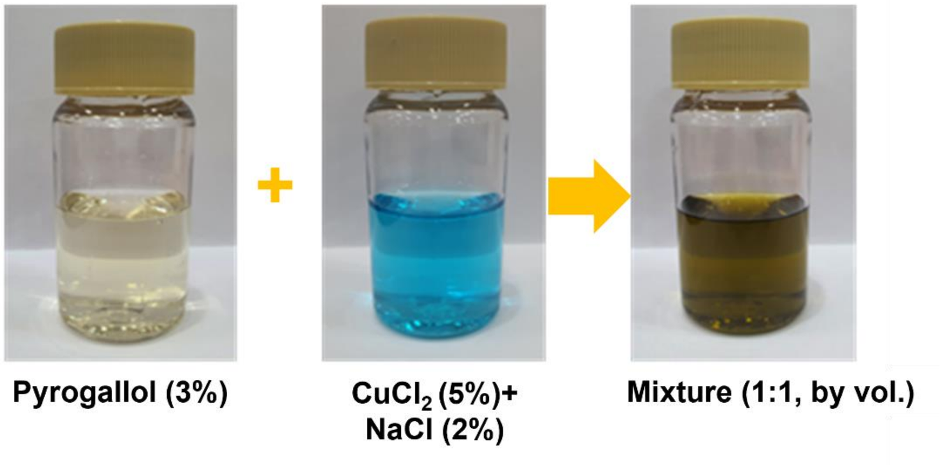

Figure S1. Photographs of Pyrogallol, $CuCl_2$ + NaCl, oligomer mixture solution mixture.

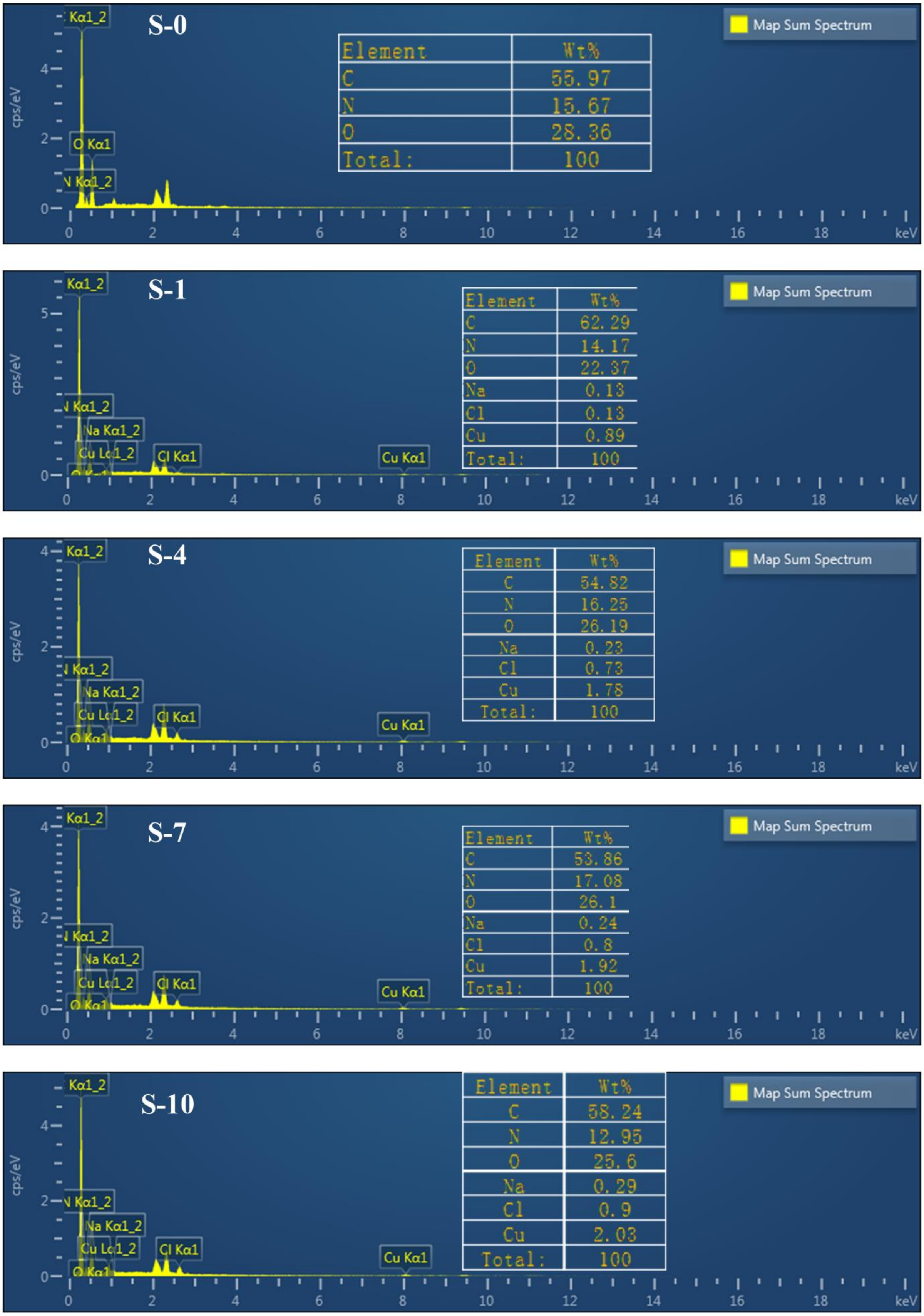

Figure S2. Elements distributed on the hair surface.

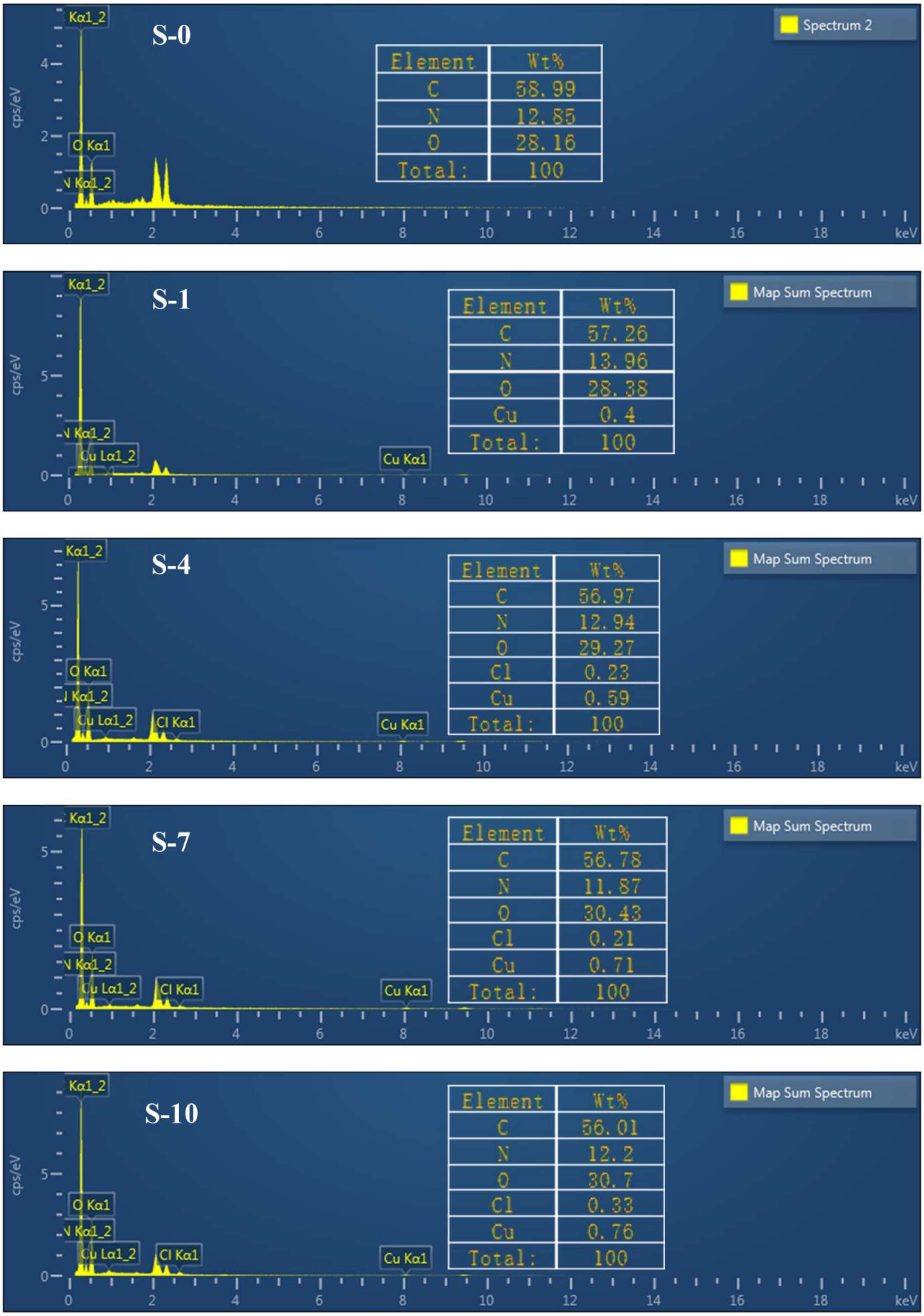

Figure S3. Elements distributed on the hair cross-section.

Table S1. Comparing thickness and tensile strength of hair dyed by commercial products and gallic acid-based dye.

Samples	Thickness (mm)		Tensile Strength (N/mm^2)	
	Before	After	Before	After
C-A	0.0557	0.045	185.79	361.5
C-B	0.056	0.039	180.9	400
C-C	0.055	0.046	186.21	391.5
C-D	0.054	0.049	179.65	408.7
C-E	0.0553	0.0511	183.78	386.1
This work	0.055	0.0652	182.79	335.32

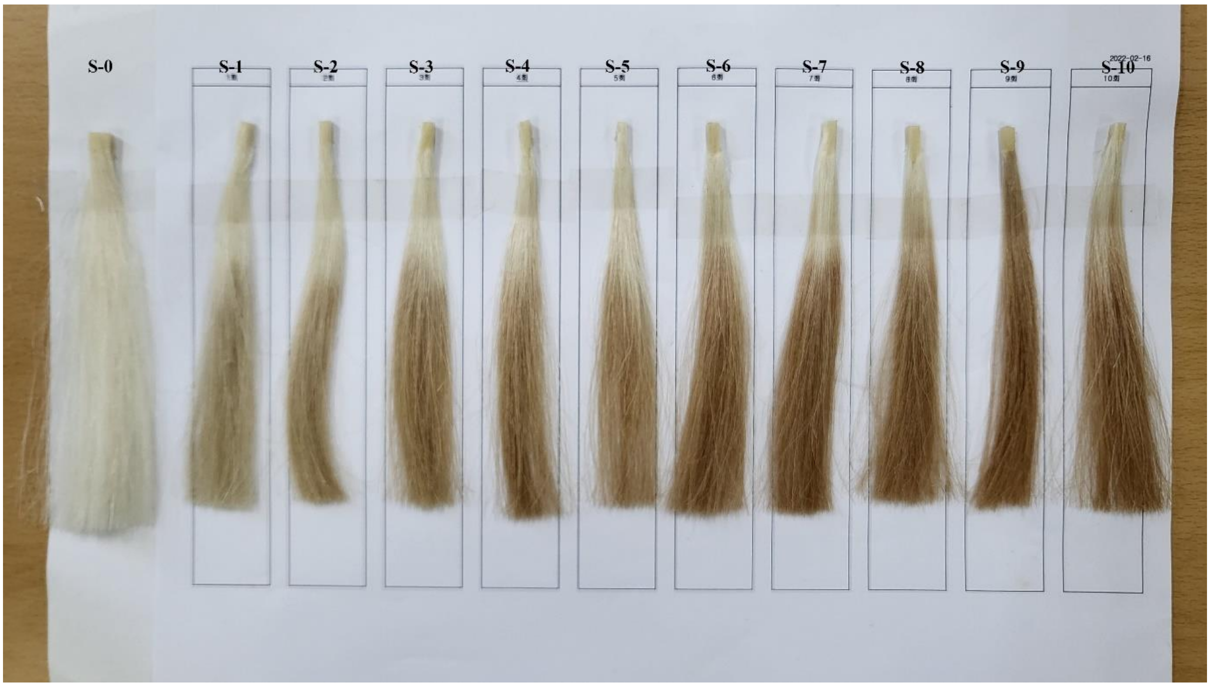

Figure S4. Photographs of the hair samples.

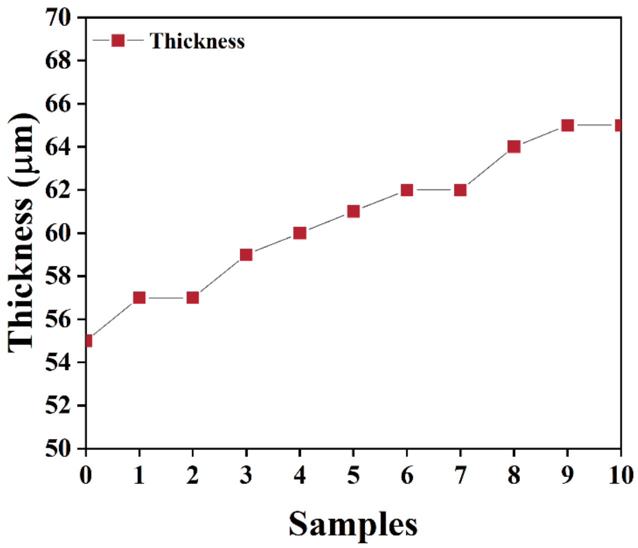

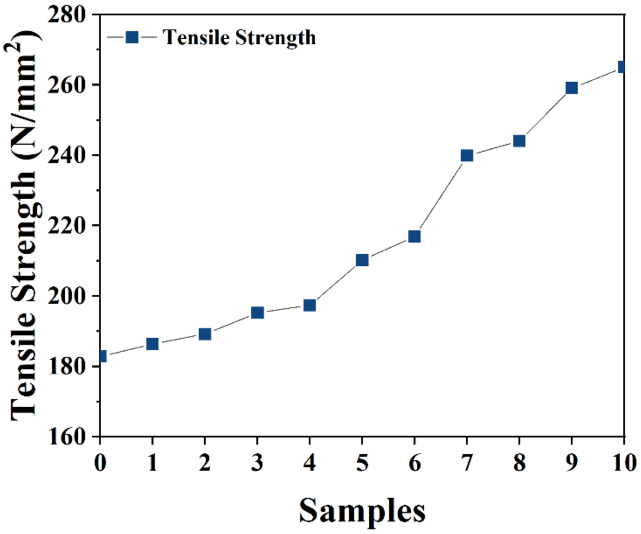

Figure S5. Thickness and tensile strength of the hair before and after the dyeing with pyrogallol.
